# Estrogen- and stress-induced DNA damage in breast cancer and chemoprevention with dietary flavonoid

**DOI:** 10.1186/s41021-016-0071-7

**Published:** 2017-02-01

**Authors:** Michiko T. Yasuda, Hiroyuki Sakakibara, Kayoko Shimoi

**Affiliations:** 1 School of Food and Nutritional Sciences, University of Shizuoka, 52-1 Yada, Suruga-ku, Shizuoka, 422-8526 Japan; 2 Faculty of Agriculture, University of Miyazaki, 1-1 Gakuen-kibanadai-nishi, Miyazaki, 889-2192 Japan

**Keywords:** DNA damage, Breast cancer, Estradiol, Estrogen metabolite, Stress, Stress hormone, Catecholamine, Cortisol

## Abstract

Breast cancer is one of the most commonly diagnosed female cancers and a leading cause of cancer-related death in women. Multiple factors are responsible for breast cancer and heritable factors have received much attention. DNA damage in breast cancer is induced by prolonged exposure to estrogens, such as 17β-estradiol, daily social/psychological stressors, and environmental chemicals such as polycyclic aromatic hydrocarbons (PAHs) and heterocyclic amines (HCAs). DNA damage induced by estrogen and stress is an important factor in the pathogenesis and development of breast cancer and is now recognized as a critical provision for chemoprevention of breast cancer. In this review, we summarize the relationships between estrogen- and stress-induced DNA damage with regard to the pathogenesis and development of breast cancer. We also discuss recent investigations into chemoprevention using dietary flavonoids such as quercetin and isoflavones.

## Background

Breast cancer comprises 18% of all female cancers and is one of the most common malignant diseases among women [1]. The mortality (rate per 100,000 populations) is reported to be 21.9 in Japan and 89.2 in the United States [1]. The etiology of breast cancer is primarily unknown, but an estimated one quarter of all breast cancers may be caused by heritable factors [2]. These include tumor suppressor genes that encode the DNA repair enzyme breast cancer susceptibility gene 1/2 (BRCA1/2), and the transcription factor p53, which targets stress response genes [3, 4]. In addition, mutations in the gene encoding the protein and lipid phosphatase and tensin homologue (PTEN), are also detected in sporadic breast cancer [5].

In addition to heritable factors, endogenous and exogenous factors levels are also thought to be involved in breast cancer. For example, prolonged exposure to estrogens, such as 17β-estradiol (E_2_), unconscious exposure to daily social/psychological stressors and environmental chemicals such as polycyclic aromatic hydrocarbons (PAHs) and heterocyclic amines (HCAs) increase the risk of breast cancer [6, 7]. DNA damage by various inducers is a well-known contributor to cancer, including breast cancer. Interestingly, estrogens and their metabolites have been shown to form DNA adducts, which damages DNA and leads to breast cancer [8]. We previously reported that oxidative DNA damage was induced in peripheral blood cells of mice exposed to social isolation stress for 7 days [9]. It was shown that stress hormones, such as glucocorticoid and catecholamines, induced DNA damage [10–12]. Interestingly, treatment with stress hormone blockers was shown to reduce the risk of death from breast cancer [13] And, an in vitro assay showed that stress hormones are potent inducers of migratory activity in breast cancer [14]. Common PAHs include benzo[a]pyrene (BaP) and benz[a]anthracen (BaA), and BaP is widely distributed in smoked and grilled meat and fish, and BaA is present in the atmosphere because of incomplete combustion of fossil fuels. These PAHs have been shown to initiate cancer (including breast cancer) by inducing DNA damage, such as the formation of DNA adducts, in various tissues [15–18]. Indeed, PAHs have been widely used to experimentally induce breast carcinogenesis [6]. HCAs are a group of mutagenic compounds that are formed during a reaction between amino acids, creatine/creatinine and sugar at high temperatures; therefore, they are found in well-cooked meats [19]. These compounds include 2-amino-1-methyl-6-phenylimidazo [4,5-*b*] pyridine (PhIP), which is a carcinogen that increases the risk of breast cancer by inducing DNA damage [20, 21]. The mechanism by which these inducers evoke breast cancer remains unknown, but DNA damage is commonly regarded as an important contributor to breast cancer.

DNA damage occurs often and is usually repaired by intracellular DNA repair mechanisms. If DNA repair mechanisms fail, then cells are programmed to undergo apoptosis. However, if apoptosis is prevented, somatic mutations continue to accumulate, leading to cancer initiation and progression [22, 23]. DNA damage has been shown to trigger various cellular responses such as DNA repair, activation of cell cycle checkpoints, and apoptosis. Activation of checkpoints delays cell cycle progression to facilitate DNA repair or eliminate damaged cells through apoptosis. Defects in these processes contribute to the initiation of cancer, and people with inherited DNA repair deficiency are predisposed to developing cancer [24, 25].

**Fig. 1:**
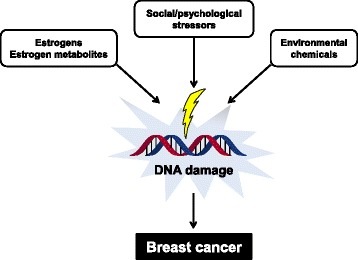
Various factors can cause DNA damage and increase the risk of breast cancer

As shown in Fig. 1, PAHs, estrogens, estrogen metabolites, physical/psychological stress induce DNA damage in breast cancer, and the accumulation can lead to an increase in breast cancer risk [26, 27]. For decades, researchers have investigated estrogen-dependent breast cancer and the underlying mechanisms have been elucidated in detail. More recently, attention has focused on how physical/psychological stresses induce DNA damage and recent reports have shown that stress alters DNA repair mechanisms [28, 29].

Stress-mediated DNA damage plays an important role in the pathogenesis and development of breast cancer in addition to estrogen-mediated DNA damage. Studying the relationship between stress-mediated and estrogen-mediated DNA damage in relation to breast cancer has been recognized as a critical provision for the chemoprevention of breast cancer. In this review, we aim to consolidate the published findings regarding the relationships between estrogen-mediated and stress-mediated DNA damage in relation to the pathogenesis and development of breast cancer. We also introduce recent findings about the chemoprevention of breast cancer with dietary flavonoids such as quercetin and isoflavones.

### Estrogen and breast cancer

Steroid hormones are important markers of breast cancer status [30, 31], particularly estrogens and their metabolites, which increase the risk of breast cancer [32]. Estrogens are endogenous sex hormones and play important roles in the development and maintenance of reproductive organs and tissue differentiation [33]. High estrogen levels in the breast are associated with an increased cancer risk in women after menopause and elevation of plasma estrogen levels was associated with breast cancer development [30, 34–36]. We previously described that the causes of estrogen-related breast cancer can be divided into two factors [37]. One is the induction of excess proliferation by estrogen receptor (ER) signaling and disrupted DNA repair resulting in the accumulation of DNA damage [38, 39]. The second is DNA damage caused by estrogen-derived metabolites modified by the cytochrome P450 (CYP) 1 family. Metabolism of estrogen is also a key factor in ER-independent carcinogenic effects [40].

**Fig. 2:**
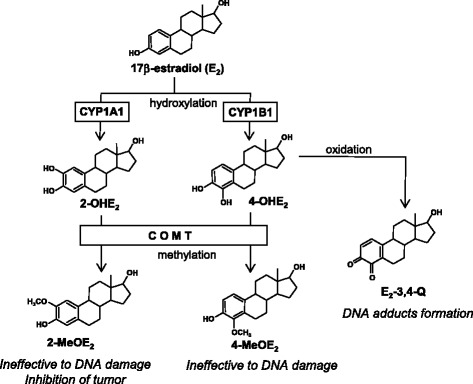
Schematic representation of typical estrogen metabolism. 17β-Estradiol (E_2_) is most common estrogen in the breast tissue. Endogenous E_2_ is hydroxylated to two types of catechol estrogens, 2-hydroxy-E_2_ (2-OHE_2_) and 4-hydroxy-E_2_ (4-OHE_2_), by Cytochrome P450 (CYP) 1A1 and CYP1B1, respectively. These catechol estrogens are further methylated to individual methoxide E_2_, 2-methoxy-E_2_ (2-MeOE_2_) and 4-methoxy-E_2_ (4-MeOE_2_), by catechol-O-methyltransferase (COMT). Methoxide E_2_ are basically ineffective compounds to DAN damage, and 2-MeOE_2_ exerts suppressive effects for tumor growth. On the other hand, 4-OHE_2_ is oxidized by any oxidative enzyme and/or metal ion, and formed E_2_-3,4-quinone (E_2_-3,4-Q), which can react with DNA directly

The most common estrogen in breast tissue is E_2_, which is mainly produced by ovarian steroidogenesis in premenopausal women. There are two pathways leading to DNA damage by E_2_: (1) the release of estrogen-DNA adducts from the DNA backbone leaving depurinated sites prone to errors in DNA repair and mutations, and (2) generation of reactive oxygen species (ROS), such as superoxide anion, produced by redox-cycling of catechol estrogens, which cause oxidative DNA damage [40]. The metabolism pathways of estrogen are extremely complex, and involve the production of many metabolites by many enzymes (Fig. 2). Endogenous E_2_ is hydroxylated to catechol estrogens 2-OHE_2_ and 4-OHE_2_ by CYP1A1 and CYP1B1, respectively [41, 42]. Catechol estrogens are then methylated by catechol-*O*-methyltransferase (COMT) to 2-methoxy-E_2_ and 4-methoxy-E_2_, respectively. 2-methoxy-E_2_ can inhibit angiogenesis and suppresses tumor growth [43]. One active metabolite is E_2_-3,4-quinone (E_2_-3,4-Q), which is produced by the oxidation of 4-OHE_2_ by any oxidative enzyme or metal ion [44, 45]. E_2_-3,4-Q can react with DNA directly and bind covalently to guanine and/or adenine, destabilizing the glycosylated bond [46–48]. In vitro and in vivo investigations have demonstrated that the unstable adducts of E_2_-metabolite, 4-OHE_2_-1-*N* ^*7*^-guanine, and 4-OHE_2_-1-*N* ^*3*^-adenine adducts, can be depurinated at the glycoside bond, creating potentially mutagenic abasic sites [35, 49–51]. Wen et al. reported that the accumulation of catechol estrogens in breast tissue in response to increased CYP1B1 and reduced COMT expression can increase breast cancer risk [52]. We previously reported that 4-OHE_2_ and COMT inhibitors can induce phosphorylated histone H2AX, a marker of DNA damage, in human MCF-7 breast cancer cells [27]. These findings support the significant role of estrogen metabolites in breast cancer development.

The reduction of estrogen quinones to hydroquinones and catechols can produce ROS, such as 8-oxo-7, 8-dihydro-2′-deoxyguanosine (8-oxo-dG), that cause oxidative DNA damage [53, 54]. Frequent errors in DNA repair can lead to the accumulation of point mutations over a long period of time. Once these mutations cause sufficient DNA damage, the risk of breast cancer is elevated [55].

### Stress and breast cancer

Stress is a state of threatened homeostasis provoked by various stressors. Physiological systems adapt to stress in various ways, including activation of the hypothalamus-pituitary-adrenal axis (HPA) as well as the sympathetic-adrenal-medullary (SAM) system, which stimulates the production of stress hormones. Prolonged or repeated activation of the HPA and SAM can interfere with their control of other physiological systems, increasing the risk of physical and psychiatric disorders [56, 57]. For several decades, studies have shown that psychological/physiological stress can contribute to the development and progression of breast cancer [12, 58–61], although the precise mechanisms have not been fully elucidated.

Case studies have shown an increased risk of breast cancer among women with a previous adverse life event or multiple stressful life changes [62–66]. Lillberg et al. investigated the relationship between stressful life events (such as divorce or separation, death of a loved one, loss of a job, increase in amount of work, interpersonal conflict, financial problems) and risk of breast cancer among 10,808 women from a Finnish Twin Cohort. They suggested a role for stressful life events in breast cancer etiology, and one of this mechanisms is associated with various hormonal secretion [67]. Animal experiments have also demonstrated the promotion of breast cancer by stress. For example, Hermes et al. and Williams et al. suggested that stress caused by social isolation increased the growth and malignancy of breast cancer tumors in rodents [68, 69]. Chronic restraint stress has also been shown to increase the rate of primary tumor growth and cause primary tumor to become more metastatic in mouse models of breast cancer [70, 71].

Disturbed circadian rhythm caused by working night shifts has been implicated as a potential risk factor for endocrine-related cancers such as breast cancer [72–74]. Figueiro et al. reported that exposure to light during the night increased cortisol levels in humans [75]. In addition, the international Agency for Research on Cancer has concluded that shift-work is potentially carcinogenic to humans [76].

One possible contributor to stress-induced cancer is the production of stress hormones, such as glucocorticoids (cortisol for humans, corticosterone for rodents) and catecholamines (adrenaline and noradrenaline), which cause damage to DNA [77, 78]. Exposure to physiological/psychological stress induces the production of stress hormones, which bind directly to the cell surface via their specific receptors. Receptor activation induces various cellular responses [79]. Okamoto et al. and Djelic et al. reported that noradrenaline induces DNA breaks and produces ROS [80, 81]. Studies in humans and animals have demonstrated that exposure to stress can contribute to DNA damage [78, 82]. We also demonstrated that stress caused by social isolation induced oxidative DNA damage in mouse peripheral blood cells using a comet assay with formamidopyrimidine DNA glycosylase (FPG). FPG has *N*-glucosidase and AP-lyase activities that are specific for oxidative DNA damage [9]. Flint et al. suggested that cortisol and catecholamines induce DNA damage and interfere with DNA repair, contributing to the transformation of murine 3T3 cells [77]. Hara et al. confirmed that chronic stress causes DNA damage via catecholamine production using animal models and cell lines [11, 83]. They suggested that activation of the β2-adrenaline receptor stimulates β-arrestin-1, activating Akt/Mdm2. Mdm2 is an E3-ubiquitin ligase and promotes the degradation of p53. Stimulation of this signaling pathway can cause DNA damage in the frontal cortex of the brain. These results provide evidence that exposure to chronic stress and continuous activation of the sympathetic nervous system can influence genomic integrity in various tissues.

We have previously reported that noradrenaline/adrenaline receptors can induce the activation of several signaling pathways in breast cancer cells to promote invasion [84]. It remains unclear whether this pathway is stimulated by DNA damage caused by catecholamine in our report. However, we have shown that DNA damage is induced by noradrenaline/adrenaline receptors in non-tumorigenic human breast cells [85], suggesting that stress and stress hormones play important roles in the initiation and development of breast cancer via DNA damage. Many studies have demonstrated that stress leads to stress hormone production, which increases the risk of breast cancer by DNA damage. However, further research is required to clarify the mechanisms underlying stress-induced breast cancer.

### Estrogen, stress, DNA damage, and cancer

The initiation and development of breast cancer can be promoted by DNA damage caused by estrogen metabolites and stress. We previously reported the synergistic effects of estrogen metabolites and stress hormones on DNA damage [85]. Combined exposure to 4-OHE2 and noradrenaline at the concentration where 4-OHE_2_ or noradrenaline alone did not cause DNA damage increased DNA damage, including the formation of AP sites and γ-H2AX in human mammary MCF-10A cells [86, 87]. Based on these findings, we suggest that co-exposure to estrogen metabolites and stress represents a novel risk factor in our daily life.

### Chemoprevention with dietary flavonoid

Diets that are rich in fruit and vegetables have been proven to decrease cancer risk [88, 89]. In addition to beneficial nutrients such as vitamins and minerals, phytochemicals such as flavonoids and other phenolic compounds contribute to these protective effects. Flavonoids are the most common polyphenolic compounds found in plants. We have previously summarized the protective effects of flavonoids against breast cancer; many flavonoids can ameliorate breast cancer by regulating the activity of CYP1 enzymes in animals and humans [37]. Endogenous E_2_ is hydroxylated to catechol estrogens by CYP1 enzymes; CYP1A1 and CYP1A2 convert E_2_ to non carcinogenic 2-OHE_2_, and CYP1B1 converts E_2_ to carcinogenic 4-OHE_2_ [41, 42]. Using ethoxyresorufin-*O*-deethylase, we have shown the effects of 18 flavonoids on CYP1 activity [90] and have summarized these findings in a previous review [37]. The double bond between the C2- and C3-positions of the C-ring means that flavones and flavonols have a tendency to selectively inhibit CYP1B1 activity rather than flavanones. The methoxy substituent on the B-ring also contributes to the strong inhibitory effects on CYP1 activity; acacetin, diosmetin, chrysoeriol, isorhamnetin, tamarixetin, and kaempferide all have strong inhibitory effects on CYP1B1 activity.

**Fig. 3:**
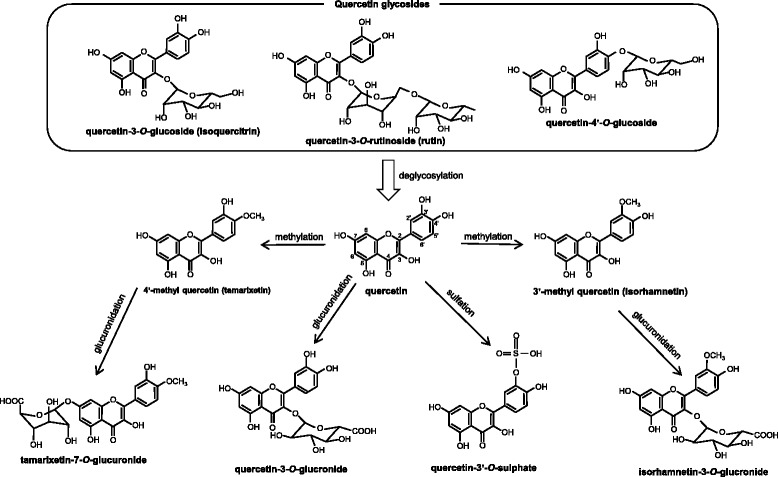
Schematic representation of typical quercetin metabolism. Quercetin is one of the major flavonoids, and present in plant foods, principally as glycosides such as quercetin-3-*O*-glucoside (isoquercitrin), −3-*O*-rutinoside (rutin) and -4′-*O*-glucoside. When consumed, quercetin glycosides are deglycosylated into their aglycone form (quercetin) by mucosal and bacterial enzymes in the alimentary canal. Subsequently, quercetin aglycone is metabolized to glucuronidated and/or sulfated derivatives (ex. Quercetin-3-*O*-glucronide and -3′-*O*-sulphate) by sulfotransferase (SULT) and uridine 5′-diphospho-glucuronosyltransferase (UGT), respectively. Additionally, quercetin aglycone is also methylated to methoxide quercetin such as tamarixetin and isorhamnetin by catechol-*O*-methyl transferase (COMT), and further glucronidated. Hece, the glucuronidated and/or sulfated derivatives may be more appropriate metabolites for quercetin aglycone for evaluation of the beneficial effects of quercetin under physiological conditions than their glycosides and aglycone form

The regulation of DNA damage is critical for the chemoprevention of estrogen- and stress-dependent breast cancer. Quercetin, including aglycone and its glycosides, is one of the most well researched dietary flavonoids, and abundant in onions. Their beneficial effects on the prevention of various diseases and bioavailability have been well studied [91–95], and we show the schematic representation of typical quercetin metabolism in Fig. 3. We have investigated the effects of quercetin and its principal metabolite quercetin-3-*O*-glucuronide, on breast cancer and found that these flavonoids can decrease 4-OHE_2_- and noradrenaline-induced DNA damage by preventing the binding of noradrenaline to the adrenaline receptor [85]. Conversely, a high-dose of quercetin is known to induce oxidative DNA damage [96, 97]. Yamashita et al. found that 10–100 μM of quercetin induced DNA damage [96] and Murota et al. reported that the major circulating forms of quercetin in human plasma (quercetin sulfate and glucuronide) are present at concentrations of 0.1–1 μM [98]. The average dietary intake of quercetin is 16 mg/day, which is a safe concentration regarding DNA damage and could prevent various diseases [96]. We have also shown that noradrenaline induces ROS production, MAPKs activation, pro-tumorigenic gene expression, and invasion in breast cancer MDA-MB-231 cells, but not in non-tumorigenic MCF-10A cells. In addition, 0.1 μM of the quercetin metabolite quercetin-3-*O*-glucuronide suppresses these responses by influencing the adrenaline receptor [84]. Following onion consumption, 1 μM of quercetin-3-*O*-glucuronide is detected in human plasma (500 g, two onions, containing quercetin glycosides equivalent to 150 mg quercetin aglycone) [98], and to our knowledge, we were the first to demonstrate the inhibitory action of quercetin-3-*O*-glucuronide at concentrations detectable in human plasma after onion consumption. Noradrenaline stimulates proliferation and promotes migration of MDA-MB-231 human breast cancer cells by activating many signaling pathways [84, 99]. Stress hormone-induced DNA damage may contribute to cancer risk. Thus, blocking interaction of noradrenaline with its receptor using quercetin metabolites may suppress various cancer-causing pathways. Taken together, these findings suggest that dietary intake of quercetin may be a powerful chemopreventive factor for estrogen- and/or stress-related breast cancer.

Phytoestrogens are compounds of plant origin with estrogen-like activity. They act as weak agonists, antagonists, or modulators of human ERs. Isoflavones are phytoestrogens and they are found in soybeans. Isoflavones such as genistein and daidzein, and the metabolite equol can bind to ERs [100] and have chemopreventive or the opposite effects on breast cancer [37, 101]. In addition, genistein modulates CYP1 expression and DNA damage in breast cells. Wei et al. suggested that physiological concentrations of genistein (5 μM) induce ROS production and stimulate breast cancer cell proliferation by inducing *CYP1B1* expression [102]. However, Leung et al. reported that oxidative DNA damage caused by PAH was inhibited by genistein (5 μM) in human breast cells by suppressing CYP1 [103]. These findings showed that genistein can both increase and decrease the risk of breast cancer. Taken together, the roles of isoflavones in breast cancer remain unclear and further research is required to elucidate them.

## Conclusion

**Fig. 4:**
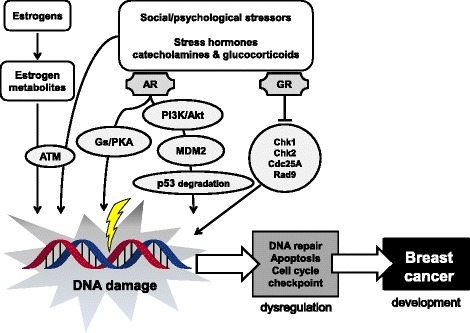
Summary of DNA damage-induced breast cancer by estrogen and stressors. Stressors induced stress hormones such as catecholamines and glucocorticoids, and these.stress hormones are recognized by each receptor, adrenaline receptor (AR) and glucocorticoid receptor (GR). These receptors activate the downstream pathways. AR stimulate the Gs/protein kinase A (PKA) pathway and phosphoinositide 3-kinase (PI3K)/Akt-mediated murine double minute 2 (MDM2), leading to p53 ubiquitination and degradation [11]. GR regulates the expression of factors critical for DNA damage signaling; Chek1 (Chk1), Chk2, cell division cycle 25 (Cdc25), and Rad9 [77]. Estrogen metabolites and noradrenaline induced DNA damage with Ataxia telangiectasia mutated (ATM) activation [27, 85]. The activation of these pathways leads to DNA damage, and accumulation of DNA damages result in induction of breast cancer

Studies have identified various risk factors for breast cancer, and many have supported the hypothesis that estrogen metabolites and stress are key risk factors for breast cancer. Although the mechanisms through which estrogens and stress contribute to breast cancer are complex, DNA damage is possibly involved. As summarized in Fig. 4, a part of these signaling pathways were investigated so far. Estrogen metabolite and stress hormone activates Ataxia telangiectasia mutated (ATM) [27, 85], and stress hormones are recognized by each receptor, followed by activation of the downstream pathways [11, 77], resulting in induction of DNA damage. Elucidating the underlying mechanisms in more detail will be of crucial importance for the development of therapeutic or preventative strategies targeting breast cancer.
